# High Performance and Low power Monolithic Three-Dimensional Sub-50 nm Poly Si Thin film transistor (TFTs) Circuits

**DOI:** 10.1038/s41598-017-01012-y

**Published:** 2017-05-02

**Authors:** Tsung-Ta Wu, Wen-Hsien Huang, Chih-Chao Yang, Hung-Chun Chen, Tung-Ying Hsieh, Wei-Sheng Lin, Ming-Hsuan Kao, Chiu-Hao Chen, Jie-Yi Yao, Yi-Ling Jian, Chiung-Chih Hsu, Kun-Lin Lin, Chang-Hong Shen, Yu-Lun Chueh, Jia-Min Shieh

**Affiliations:** 1 National Nano Device Laboratories, No. 26, Prosperity Road 1, Hsinchu, 30078 Taiwan; 2 Department of Materials Science and Engineering, National Tsing Hua University, Hsinchu, 30013 Taiwan; 3 Departments of Photonics and Institute of Electro-Optical Engineering, National Chiao-Tung University, Hsinchu, 30010 Taiwan

## Abstract

Development of manufacture trend for TFTs technologies has focused on improving electrical properties of films with the cost reduction to achieve commercialization. To achieve this goal, high-performance sub-50 nm TFTs-based MOSFETs with ON-current (I_on_)/subthreshold swing (S.S.) of 181 µA/µm/107 mV/dec and 188 µA/µm/98 mV/dec for NMOSFETs and PMOSFETs in a monolithic 3D circuit were demonstrated by a low power with low thermal budget process. In addition, a stackable static random access memory (SRAM) integrated with TFTs-based MOSFET with static noise margins (SNM) equals to 390 mV at V_DD_ = 1.0 V was demonstrated. Overall processes include a low thermal budget *via* ultra-flat and ultra-thin poly-Si channels by solid state laser crystallization process, chemical-mechanical polishing (CMP) planarization, plasma-enhanced atomic layer deposition (ALD) gate stacking layers and infrared laser activation with a low thermal budget. Detailed material and electrical properties were investigated. The advanced 3D architecture with closely spaced inter-layer dielectrics (ILD) enables high-performance stackable MOSFETs and SRAM for power-saving IoT/mobile products at a low cost or flexible substrate.

## Introduction

Thin film transistors (TFTs) are commonly used in large-area and flexible electronics, such as displays, biosensors, phototransistors and memories. The development trend for TFTs technologies has been focused on improving electrical properties of films and the cost reduction to achieve commercialization. Amorphous Si (a-Si) has been used as the active layer of TFTs over the past few decades. However, the low mobility (~0.1 cm^2^/V-s) with the poor stability limits the device performance. To overcome such issues, devices based on polycrystalline Si (poly-Si) or amorphous oxide semiconductors (AOSs) have been utilized and investigated^1–5^. AOSs are capable of transparent and flexible TFTs because of its excellent optical transparency and low-temperature process. Nonetheless, a relatively low mobility (~10 cm^2^/V-s) and poor stability are still challenges. The low-temperature poly-Si (LTPS) has a number of advantages, including the relatively high mobility (10–100 cm^2^/V-s) with excellent stability^6^.

Integration of complementary metal oxide semiconductor (CMOS) circuits comprising both p- and n-type TFTs are basic building blocks for complex integrated circuits toward system-on-chip and other electronic applications. To achieve this goal, heterogeneously integrated three-dimensional integrated circuit (3DIC) technology is the best candidate to achieve this target to realize device integration with high performance, multifunction, wide bandwidths and low power consumption. To date, 3DIC from Through-Silicon-Via (TSV) technology developed by IC manufacturing company has been demonstrated (Figure S1a), providing a higher density with less parasitic loading, while large dimension with a long connect distance, as well as significant parasitic capacitance compared to typical via/contact in CMOS process, have to be taken into account. Alternatively, the monolithic 3D-IC through layer by layer stacking process has been proposed (Figure S1b), providing advantages of high density, vertical interconnection, low cost and high yield. However, the challenge of the monolithic 3D-IC^7–13^ is on how to reduce thermal impact of device fabrication to avoid degradation of pre-existing devices, resembling back-end process.

In this regard, we demonstrated a polycrystalline Si thin-film transistors (TFTs) based on the monolithic 3D-IC sequential integration (3DSI) method to achieve a low-cost fabrication process with a low thermal budget for the monolithic 3D-IC application. High-performance TFTs based on MOSFETs with a sub-50 nm gate length (L_G_)/gate width (W_G_) and a stackable static random access memory (SRAM) were developed and demonstrated through the monolithic 3D circuits by introducing (1) low thermal budget of ultra-flat and ultra-thin poly-Si channels by a solid state laser crystallization process, chemical-mechanical polishing (CMP) planarization, (2) plasma-enhanced atomic layer deposition (ALD) gate stacking layers and (3) infrared laser activation with a low thermal budget. The advanced 3D architecture with a closely spaced interlevel dielectric (ILD) enables high-performance stackable MOSFETs and SRAM for the power-saving internet of things (IoT)/mobile products on low cost or flexible substrate.

## Results and Discussion

**Figure 1:**
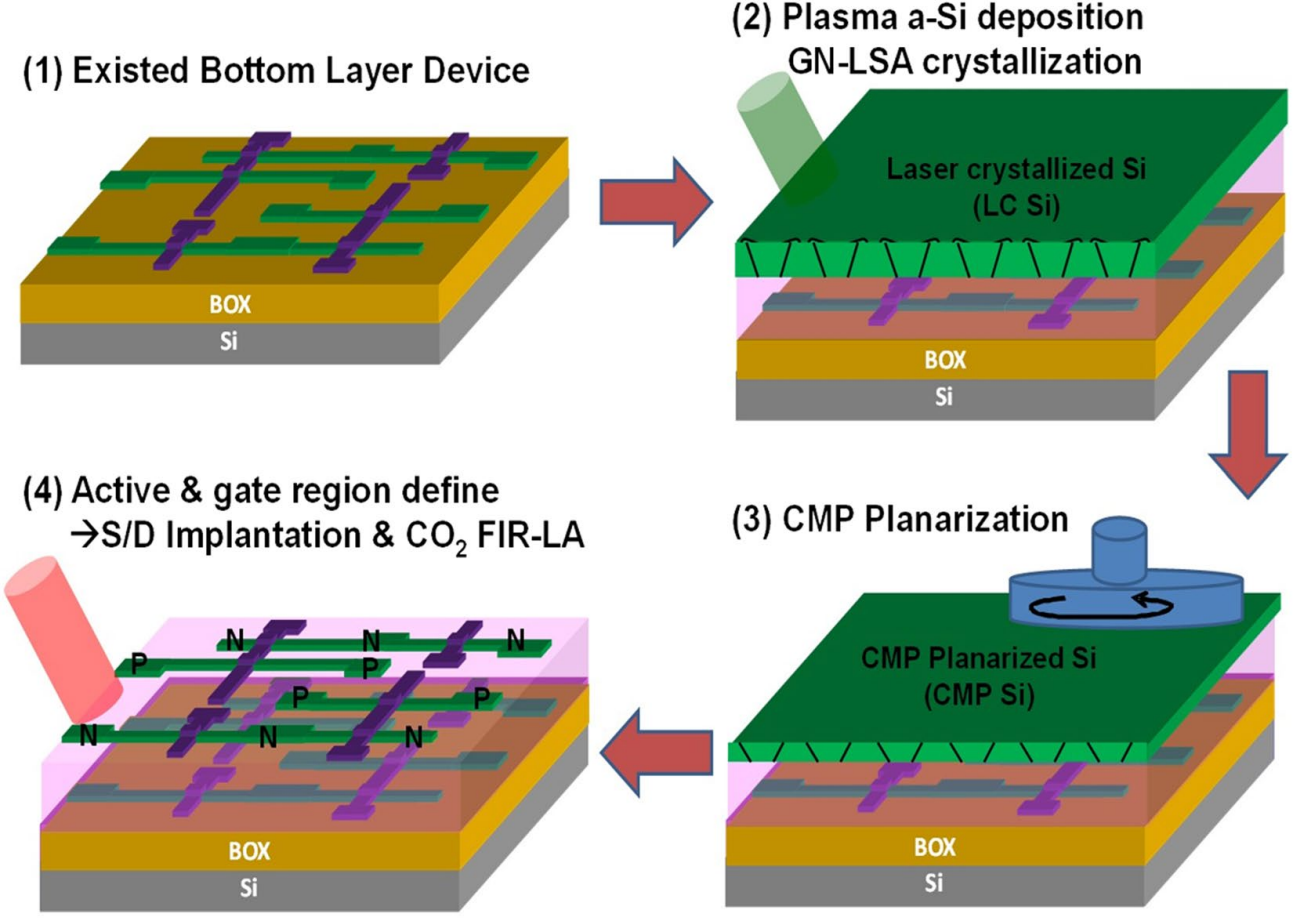
Schematics and process flows of low cost and low thermal budget monolithic 3D IC: (1) prefabrication of bottom layer devices, (2) deposition of an a-Si film followed by a laser crystallization process, (3) a chemistry mechanical publishing (CMP) process and (4) active/gate region via source/grain (S/D) implantation followed by activation of implants by a CO_2_ laser annealing process.

Low-temperature crystallization processes of the poly-Si channel films have been studied extensively, including solid-phase crystallization (SPC), laser crystallization (LC) and metal-induced lateral crystallization (MILC)^14–16^. The MILC method has a metal contamination issue in the poly-Si channel, resulting in the degradation of the junction leakage. The conventional SPC process needs a higher temperature (~600 °C) with a longer annealing period (~24 hours) to achieve the high device quality. Therefore, laser crystallization (LC) is the most commonly used to produce a poly-Si film with a low defect density and a higher field effect mobility, which was utilized to achieve the poly-Si film from the crystallization of the a-Si film in our study^15^. Figure 1 schematically illustrates overall fabrication steps of MOSFETs with ultra-thin and -flat poly-Si films through the monolithic 3D process (See detailed experimental section in Supplementary Information), including (1) prefabrication of bottom layer devices, (2) deposition of an a-Si film followed by a laser crystallization process, (3) a chemistry mechanical publishing (CMP) process and (4) active/gate region *via* source/grain (S/D) implantation followed by activation of implants achieved by a CO_2_ laser annealing process. Note that the surface roughness is a critical issue after the laser crystallized poly-Si process, resulting in the blistering effect because of the residual interior hydrogen during the laser annealing. However, other factors such as a long laser pulse with high energy may trigger the melting process of materials, resulting in rough surface, and deteriorates carrier mobility because of the electron scattering^17, 18^. Therefore, the chemical-mechanical planarization (CMP) process will be used to reduce the surface roughness. In addition, the poly grain morphologies, such as grain size, grain crystallinity, internal stress and grain orientation as well as defect density after the laser crystallization process are important factors to influence device performance.

**Figure 2:**
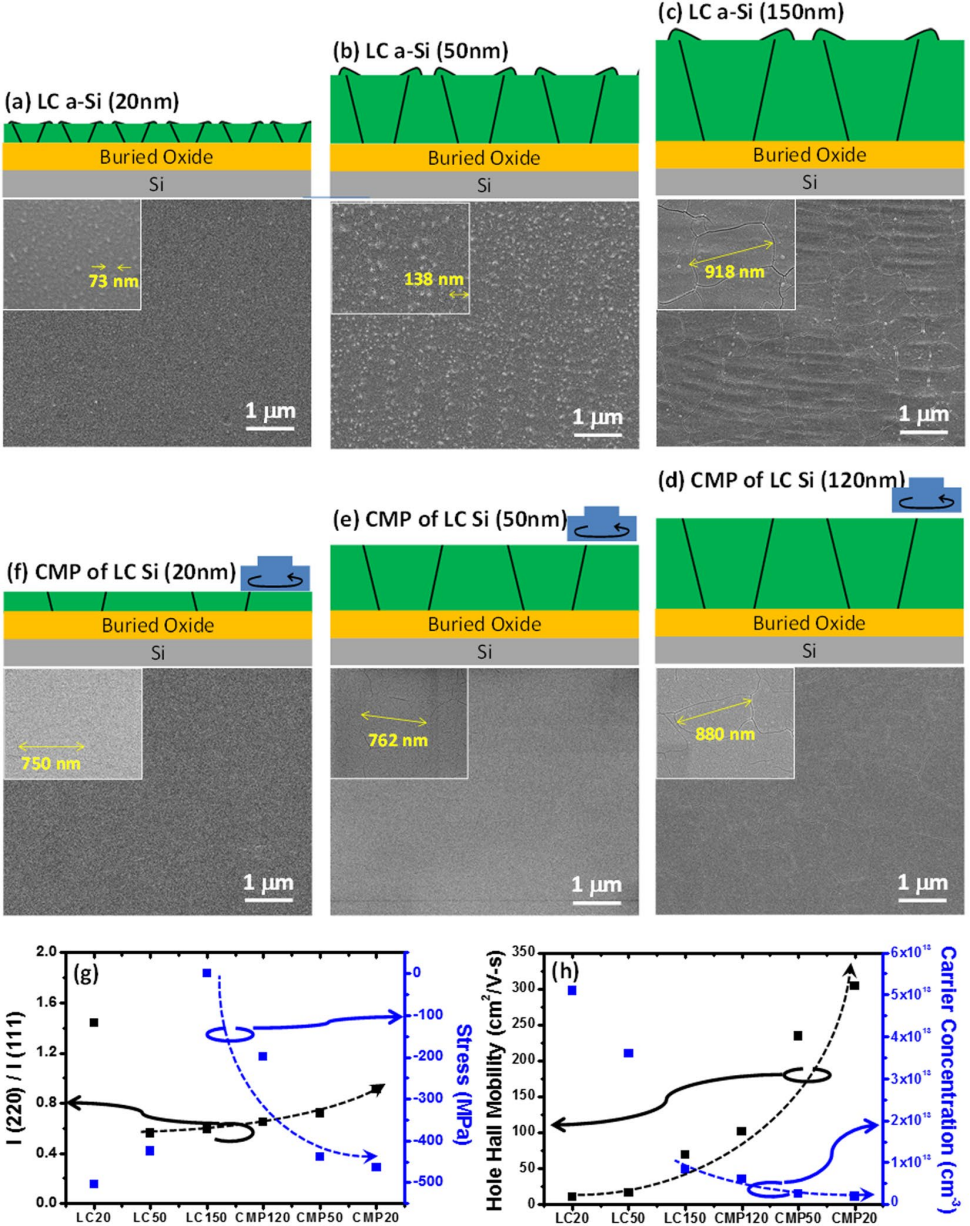
(**a**–**c**) Top view morphologies and cross-sectional schematics of laser crystallized poly-Si thin films at different original a-Si thickness. Insets show the typical grain size with high magnification. (**d**–**f**) Top view morphologies and cross-sectional schematics of poly-Si thin films after the CMP planarization from the original a-Si thickness of 150 nm. Insets show the typical grain size with high magnification. (**g**) Intensity ratio of (220)/(111) planes and stress for different crystallized poly-Si thin films. (**h**) Bulk mobility of carrier concentrations for different crystallized poly-Si thin films.

To optimize the laser induced crystallization, a-Si thin films with different thicknesses of 20, 50 and 150 nm were deposited by the high density plasma chemical vapor deposition (HDP-CVD), followed by the laser crystallization to obtain the crystallized poly-Si films marked as LC20, LC50 and LC150 nm, respectively as shown in Fig. 2(a) to (c). The corresponding schematics are shown in the top region in Fig. 2(a) to (c). Note that to define the grain size clearly, crystallized poly-Si films were then Seeco etched (solution of K_2_Cr_2_O_7_ water mixed with HF) first to enhance the contrast between grains and grain boundaries. As a result, average grain sizes can be determined by scanning electron microscope (SEM) to be ~73, ~138, and ~918 nm for LC 20, 50 and 150 nm as shown in insets of Fig. 2(a) to (c) with the average surface roughness of ~8.01 ~5.27 and ~6.21 nm confirmed by an atomic force microscope (AFM) as shown in Figure S2(a) to (c), respectively. Clearly, grains grow as the thickness of crystallized poly-Si films increases, which is consistent with the report from the literature^19^. To fulfill sub-50 nm-thick MOSFET with the improved current drivability and the suppressed short channel effect (SCE), the CMP planarization methodology was used to thin down the thickness of the crystallized poly-Si thin films from 150 nm into 120 nm, 50 nm and 20 nm marked as CMP 120 nm, CMP 50 nm and CMP 20 nm as shown in Fig. 2(d) to (f), respectively. The average grain size, surface roughness, crystalline ratio of (220)/(111), internal stress, Hall mobility and carrier concentration with crystallized poly-Si thin films before and after CMP treatment are listed in Table 1. Clearly, average grain sizes are slightly reduced to 899.0 nm, 762.0 nm and 749.3 nm after the CMP planarization with thicknesses of 120 nm, 50 nm and 20 nm, respectively. The average roughness is also greatly reduced into 2.03 nm, 1.14 nm and 0.50 nm, respectively (Figure S2c to e). Surface orientations for all crystallized poly-Si films were extracted by X-ray diffraction analysis (Figure S3) where three peaks located at 28.5°, 47.4° and 56.3° represent (111), (220) and (311) planes, respectively. We further calculate the residual strain for all crystalized poly-Si films with different thicknesses before and after the CMP planarization process from X-ray diffraction analysis where the 150 nm-thick crystalline poly-Si film (LC 150 nm) was used a reference for comparison as shown in Table S1. The detailed residual strain calculation has been mentioned in experimental part and Supplementary Information in Figure S4. The corresponding intensity ratios of (220)/(111) and internal stress were plotted in Fig. 2(g) and summarized in Table 1 where a minus sign represents the compressive stress. The trend of preferred orientation changes from (111) to (220) as the thickness of the crystallized poly-Si thin film decreases, which is probably due to the preferred grain orientation initially grown from (220) rather than (111) in the laser crystallized seed layer^20, 21^. In addition, the compressive residual stress distinctly increases with the thinner crystalline poly-Si after the CMP planarization because of a large thermal conductivity difference between SiO_2_ (1.5 W/m-K) and a-Si (~34 W/m-K). The residual compressive stress related to devices performance will also be discussed later. The findings indicate that ultra-thin and ultra-flat crystallized poly-Si thin films with the larger grain size can be achieved by combining the laser crystallization of the a-Si film, followed by the CMP planarization process.

**Table 1:** Material properties of laser crystallized a-Si thin films and CMP planarized poly-Si thin films.

	Grain Size (nm)	Roughness (nm)	I(220)/I(111)	Stress (MPa)	Hall Mobility (cm^2^/V-s)	Carrier Concentration (cm^−3^)
20 nm	68.0 ± 4.1	8.01	1.44	−504.2	10	5.1 × 10^13^
LC 50 nm	123.5 ± 17.4	5.27	0.56	−424.6	16	3.6 × 10^13^
LC 150 nm	968.5 ± 71.9	6.21	0.59	0	69	8.4 × 10^12^
CMP 120 nm	899.0 ± 18.3	2.03	0.65	−119.4	101	6.0 × 10^12^
CMP 50 nm	752.1 ± 74.1	1.14	0.72	−437.9	234	2.5 × 10^12^
CMP 20 nm	749.3 ± 52.7	0.50	0.91	−464.4	305	1.9 × 10^12^

Moreover, to evaluate the intrinsic qualities of crystalline poly-Si thin films, bulk mobility and carrier concentrations normalized to thickness were measured by Hall measurements as shown in Fig. 2(h) and summarized in Table 1 where a P-type semiconductor behavior can be confirmed due to acceptor-like type traps existed at the grain boundary^19^. Obviously, the carrier concentration was found to be greatly reduced from LC 20 nm, LC 50 nm to LC 150 nm owing to the decrease of grain boundary portions with the increased grain size and is still larger than 8.4 × 10^12^ cm^−3^ (Table 1). Reduction of carrier concentrations from 6 × 10^12^ to as low as 1.9 × 10^12^ cm^−3^ was observed after the CMP planarization once thicknesses of crystalline poly-Si thin films were reduced from 150 nm to 120 nm, 50 nm and 20 nm, respectively. The carrier mobility, which is inversely proportional to carrier concentration, *σ* = *q*(µ_*n*_ *n* + µ_*p*_ *p*), can be extracted as shown in left-hand side of Fig. 2(h). Distinctly, the extracted bulk mobility can be improved to the thinner poly-Si thin film after the CMP planarization, while the maximized extracted bulk mobility of 305 cm^2^/V-s for the CMP planarized poly-Si thin film with the thickness of 20 nm can be found owing to the significant suppress of surface roughness scattering.

**Figure 3:**
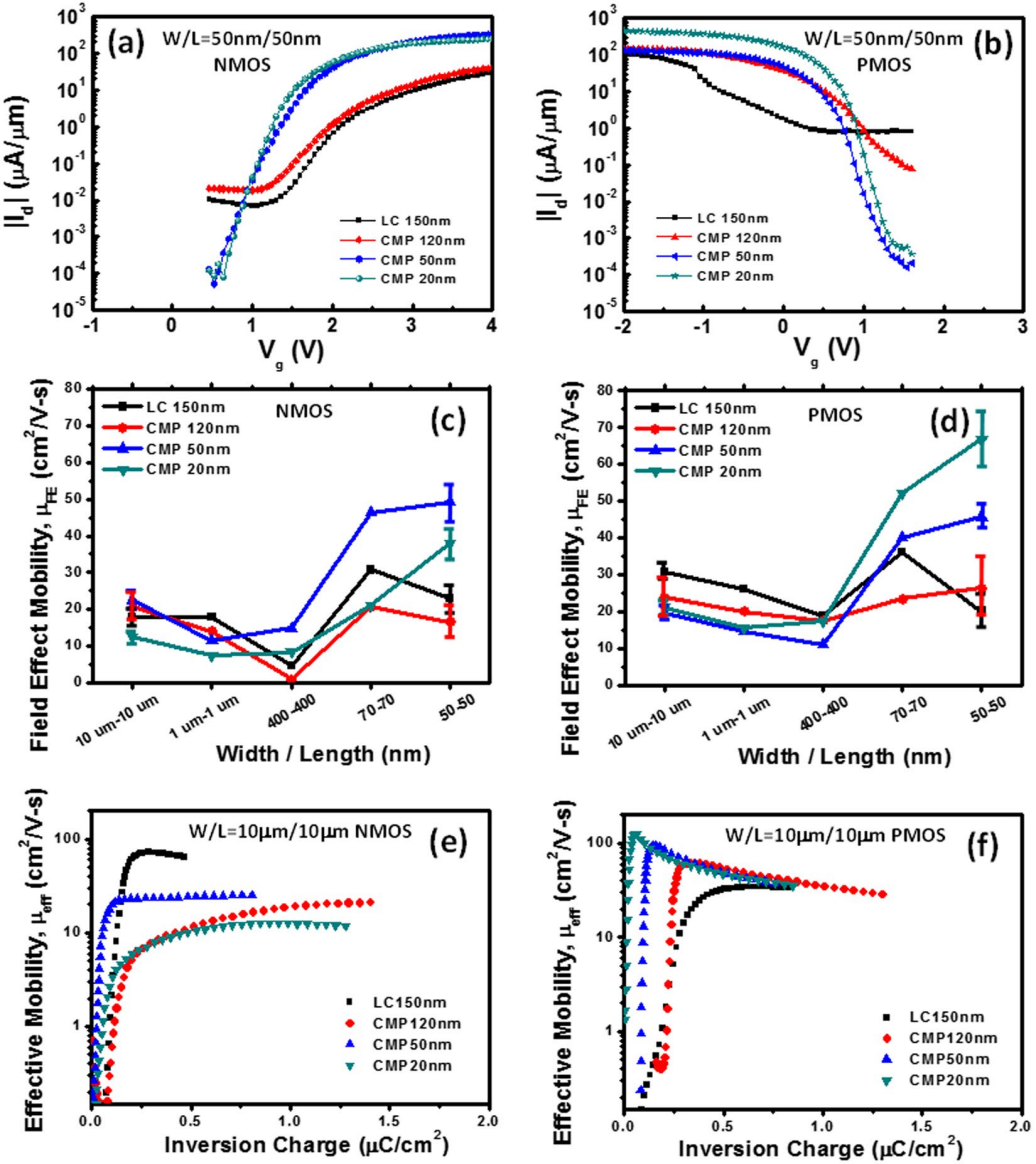
(**a** and **b**) I-V behaviors of NMOSFETs and PMOSFETs with the channel width/length (50 nm/50 nm) at different crystallized poly-Si thin films as channel layers. (**c** and **d**) Field effect mobility (**m**_FE_) of NMOSFETs and PMOSFETs as the function of channel lengths at different crystallized poly-Si thin films as channel layers. (**e**,**f**) Effective mobility (**m**_eff_) of NMOSFETs and PMOSFETs with a long channel width/length of 10 mm/10 mm at different crystallized poly-Si thin films as channel layers.

To exam ultra-thin and -flat poly-Si thin film transistors, the crystallized poly-Si films, LC 150 nm, CMP 120 nm, CMP 50 nm and CMP 20 nm were selected to proceed device fabrication processes for comparison. Moreover, to demonstrate the implementation of deep sub-100 nm TFT-based MOSFETs, the channel width (W)/channel length (L) of 50 nm/50 nm was fabricated with different poly-Si channel thicknesses as shown in Fig. 3 for comparison. Figure 3(a) show I-V behaviors of TFT-based NMOSFETs at V_d_ = 1 V with the W/L of 50 nm/50 nm (1/10 times of poly-Si grain size) and extracted I-V results were summarized in Table 2. The conventional TFTs with the channel width/length of 10 µm/10 µm is shown in Figure S5(a) and the corresponding I-V behaviors at V_d_ = 1 V are also summarized in Table S2. Threshold voltage (V_th_) is defined as the gate voltage, at which the drain current reaches to 0.1 µA/µm at V_d_ = 1.0 V and on current is defined as the drain current (I_d_), at which the gate voltage equals to V_th_ + 0.8 V. For sub-50 nm devices (Fig. 3a), both subthreshold swing (S.S.) and on current (I_on_) are largely improved from 415 mV/dec and 8.0 µA/µm for the LC 150 nm-thick crystallized poly-Si film to 151 mV/dec and 67.0 µA/µm for the CMP 20 nm-thick crystallized poly-Si film because of the suppressed surface roughness scattering after the CMP planarization. Interface trap state density (N_it_) can be extracted from S.S. by *N*_*it*_ = [(*SS*/ln*10*)*(*q*/*kT*) − *1*]*(*C*_*ox*_/*q*)^22^. As a result, the decrease of N_it_ from 45.1 × 10^12^ cm^−2^ for the LC 150 nm-thick crystallized poly-Si film to 11.6 × 10^12^ cm^−2^ for the CMP 20 nm-thick crystallized poly-Si film can be achieved, which further reduce the V_th_ from 1.59 V to 1.05 V for the CMP 20 nm-thick crystallized poly-Si film because V_th_ is significantly reduced by decreasing concentrations of traps after the reduction of the poly-Si channel thickness^19^. In addition, on/off ratio (I_on_/I_off_) > 1 × 10^6^ can be found for CMP 20 nm- and CMP 50 nm-thick crystallized poly-Si films, while the CMP 50 nm-thick crystallized poly-Si film provides a much higher field effect mobility of 49.3 cm^2^/V-s. The TFT-based PMOSFETs at V_d_ = −1.0 V with W/L = 50 nm/50 nm and 10 µm/10 µm were also measured in Figs 3(b) and S5(b), and the corresponding device results are summarized in Table 2 and Table S2. Clearly, the device performance from the LC 150 nm-thick crystallized poly-Si film exhibits nearly punch through phenomenon owing to short channel effect because of acceptor-like type traps, while improved S.S and on-current of 119 mV/dec, 128 mV/dec and 166.0 µA/µm, 58.8 µA/µm for CMP 20 nm- and CMP 50 nm-thick crystallized poly-Si films can be measured, respectively, yielding the high I_on_/I_off_ > 5 × 10^6^ with the extracted field effect mobility (µ_FE_) of 66.7 and 45.6 cm^2^/V-s for CMP 20 nm and CMP 50 nm crystallized poly-Si films, respectively. In addition, the reduced N_it_ was measured from 36.7 × 10^12^ cm^−2^ to as low as 7.6 × 10^12^ cm^−2^ with a reduced V_th_ from 1.47 V to 1.00 V for the CMP 20 nm-thick crystallized poly-Si film. It is found that much poor device results were measured for the conventional TFTs with the W/L of 10 µm/10 µm even for the crystallized ploy-Si films after the CMP planarization (CMP 120 nm-, CMP 50 nm- and CMP 2 nm-thick crystallized ploy-Si films) (Figure S5a and b) because of the large electron scattering caused by grain boundaries^23^. Thus, the effect of the CMP planarization is prominent for the improved device performance as the channel length < the grain size.

**Table 2:** Parameters of channel width/length (50 nm/50 nm) for NMOSFETs and PMOSFETs as the function of channel thicknesses.

NMOSFET W/L = 50 nm/50 nm	S.S. (mV/dec)	V_th_ (V)	I_on_ (uA/um)	N_it_ (cm^−2^)	μ_FE_ (cm^2^/V-s)
LC 150 nm	415	1.59 ± 0.39	8.0	45.1 × 10^12^	23.0 ± 5.4
CMP 120 nm	388	1.48 ± 0.23	5.6	41.6 × 10^12^	16.6 ± 6.3
CMP 50 nm	172	1.05 ± 0.35	51.2	14.3 × 10^12^	49.3 ± 7.3
CMP 20 nm	151	1.05 ± 0.16	67.0	11.6 × 10^12^	38.0 ± 6.0
**PMOSFET W/L = 50 nm/50 nm**	**S.S. (mV/dec)**	**V**_**th**_ **(V)**	**I**_**on**_ **(uA/um)**	**N**_**it**_ **(cm** ^**−2**^ **)**	**μ**_**FE**_ **(cm** ^**2**^ **/V-s)**
LC 150 nm	N/A	N/A	N/A	N/A	20.1 ± 6.5
CMP 120 nm	349	1.47 ± 0.36	12.1	36.7 × 10^12^	26.3 ± 11.5
CMP 50 nm	128	0.94 ± 0.38	58.8	8.7 × 10^12^	45.6 ± 4.7
CMP 20 nm	119	1.00 ± 0.32	166.0	7.6 × 10^12^	66.7 ± 10.9

Furthermore, Fig. 3(c) and (d) exhibit extracted field effect mobility (µ_FE_) of NMOSFETs and PMOSFETs with varied device sizes. Note that the maximum µ_FE_ was measured, while devices size is below 70 nm, especially below 50 nm because the average grain size is much larger than device size in such conditions and the grain boundary impact could be excluded, yielding high carrier mobility and low SS. However, as shown in Table 2, the mobility fluctuation of small device size (50 nm/50 nm) in thinner film slightly increases compared to larger device size (10 µm/10 µm) in thicker film. This is due to large device size across multiple grains, leading to an averaged mobility, while the small device size device depends on the orientation of single grain or stress, resulting in a specific mobility. However, the CMP process can compensate such effects by providing a flat and thin channel to avoid scattering effect and achieve well gate control capability, especially in small size devices, which explains steeper subthreshold swing, higher on currents and lower V_th_ with better uniformity, especially in CMP 20 nm condition. Moreover, in the conventional TFTs structure, the presence of numerous long grains in the channel width with different crystal orientations may statistically reduce the mobility^18^. It is why the minimum of µ_FE_ with the poor carrier mobility was found for the channel widths of 400 nm and 1 µm because of the comparable scale between the channel width and grain size. Moreover, regarding the on-current and sub-threshold swing fluctuation, the smaller fluctuation with the CMP process with 2*σ* = 95.4%, around 50% improvement, can be achieved. To evaluate inherent channel mobility of MOSFETs, effective mobility is widely used. The effective mobility (µ_eff_) is deduced from the first-order one-dimensional model in the linear model at V_d_ = 50 mV given by µ_*eff*_ = (*L*/*W*)(*g*_*D*_/*C*_*ox*_(*V*_*g*_ − *V*_*th*_)) = (*L*/*W*)(*g*_*D*_/*Q*_*inv*_) where I_d_ is the drain current, L is the length, W is the width of the channel, V_g_ is the gate voltage, V_th_ is the threshold voltage, Q_inv_ is the inversion charge and *g*_*D*_ is drain conductance given by *g*_*D*_ = *d I*_*d*_/*d V*_*d*_ *|*_*Vg*=*const*._, respectively^24^. Normally, the capacitance-voltage (C-V) was conducted to accurately extract Q_inv_ by measuring the gate-to-channel capacitance as the function of the gate voltage, especially in a thick SiO_2_ gate dielectric structure^25^. However, it is not sufficient to evaluate the Q_inv_ for the advanced high-k gate dielectric layer owing to high concentrations of interface traps, leading to high leakage current. In order to obtain the accurate Q_inv_, calibration methodologies, including high frequency (100 kHz) split C-V method to correct overestimation of Q_meas_ (=Q_inv_ + Q_trap_) as interface traps, thick enough (5 nm) high-k dielectric layer to reduce leakage current and silicide process to reduce contact resistance, were introduced during the device fabrication^26^. The corresponding effective mobility as the function of inversion charge results for NMOSFET and PMOSFET with LC150 nm-, CMP 120 nm-, CMP 50 nm-, and CMP 20 nm-thick crystallized ploy-Si films are shown in Fig. 3(e) and (f), respectively. The extracted effective mobility as the function of gate voltages is also shown in Figure S6. Obviously, the maximum effective mobility occurs near the threshold voltage and saturate in an inversion charge >0.2 µC/cm^2^ due to the coulomb scattering of carriers induced by interface traps^26, 27^. Note that the effective mobility of NMOSFET is larger than that of PMOSFET in the LC 150 nm-thick crystallized poly-Si film because the inherent electron mobility is larger than the hole mobility owing to the different effective mass. The hole effective mobility increases with an increase in the crystallized poly-Si film thickness after the CMP planarization, while the electron effective mobility decreases with the decrease in the crystallized poly-Si film thickness due to the compressive residual stress (Table 1). In addition, the residual compressive stress is also beneficial to the increase of the hole mobility^28^.

**Figure 4:**
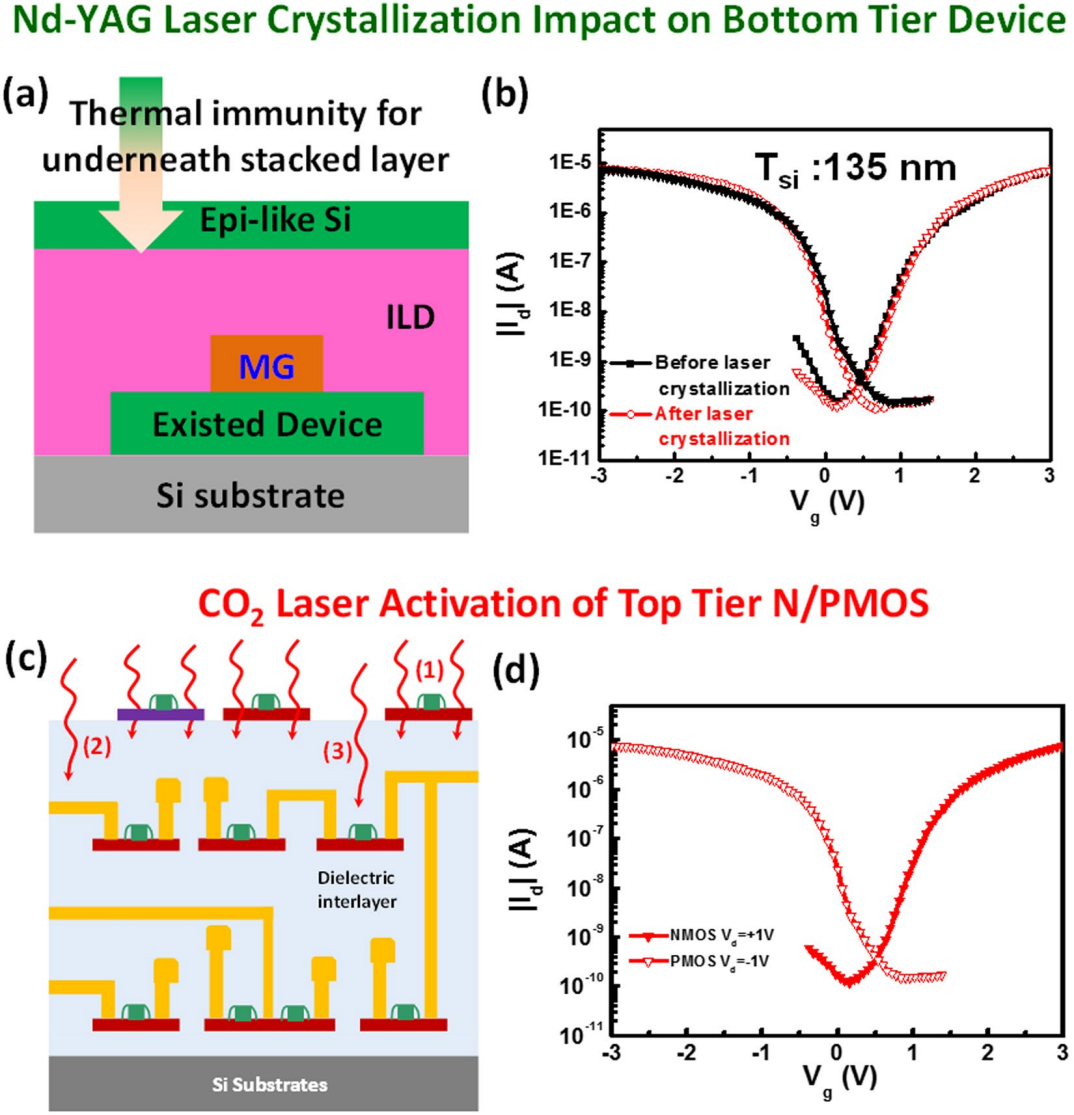
(**a**) A schematic of top tier laser crystallization on bottom tier device. (**b**) The corresponding I_d_-V_g_ behaviors of the a-Si thin film with a critical thickness >135 nm before and after the top tier laser crystallization. (**c**) A schematic of interactions between CO_2_ laser and devices, including top tier source/drain regions effective activation, bottom tier metallization reflection and defects repair of bottom tier channels. (**d**) The corresponding I_d_-V_g_ behaviors of top tier device after the activation process by the CO_2_ laser.

To realize the compatibility of the monolithic 3D-IC sequential integration process, the process temperature was evaluated in this section. For the back-end metallization, the sustainable temperature should be below 400 °C. To achieve this goal for the monolithic 3D-IC sequential integration, a Nd:YAG laser with a wavelength of 532 nm, which is the fastest rapid heating process to replace rapid thermal annealing (RTA) or flash lamp annealing process in the conventional CMOS process, was used for a top layer heating process to achieve the crystallized poly-Si film from the a-Si film because of an excellent absorption coefficient of ~10^4^ cm^−1^ at a wavelength of 532 nm^29^. The schematic of the laser crystallization process is shown in Fig. 4(a). The high-intensity laser beam causes the nearly complete melting (also denoted as partial melting) and a small amount of residual unmolten Si acts as a liquid/solid interface crystal seed, permitting controlled growth upward from the interface with the molten Si^14^. The tight control of the laser flux at 320 mJ/cm^2^ was found to precisely achieve the epitaxial-like (e-like) Si with the best crystallization from a-Si. A critical top a-Si thin film with a thickness of 135 nm was prepared on existing bottom tier NMOSFETs and PMOSFETs, followed by the rapid laser crystallization process in order to demonstrate the process compatibility. Figure 4(b) shows I_d_-V_g_ characteristics of the bottom tier NMOSFETs and PMOSFETs before and after the laser crystallization processes. Clearly, no change from I_d_-V_g_ behaviors before and after the laser crystallization process confirms that the crystallization of the e-like Si from a-Si by the fastest laser crystallization process is a very stable and compatible process. In addition, a CO_2_ far-infrared laser annealing technology was used for a dopant activation process without causing device degradation because of the low thermal budget (400 °C) where the laser energy is completely absorbed by defect-related free carriers then transferring energy to trigger lattice vibration, resulting in diffusion of dopants to Si site (Figure S7)^12, 30–32^. The corresponding schematic of the CO_2_ laser annealing process is shown in Fig. 4(c) where top tier MOSFETs are directly irradiated by the CO_2_ laser to activate dopants in source/drain regions, and the CO_2_ laser can be highly reflected by TiN or TaN metal gate to avoid damaging tier MOSFETs because of inherent high reflectivity of a long wavelength that is compatible with the gate first process. The corresponding I-V behaviors of top tier devices after the activation of the dopants by the CO_2_ laser are shown in Fig. 4(d) where SS and V_th_ of 212, 171 mV/dec and 0.89, 0.05 V for NMOSFET and PMOSFET were measured, respectively. In addition, the CO_2_ laser also enable effective activation a 8 inch wafer with nearly identical transfer characteristics, indicating the uniformity of the CO_2_ laser activation process where four devices from NMOSFETs and PMOSFETs on the 8 inch wafe exhbit the identical I-V behaviors (Figure S8).

**Figure 5:**
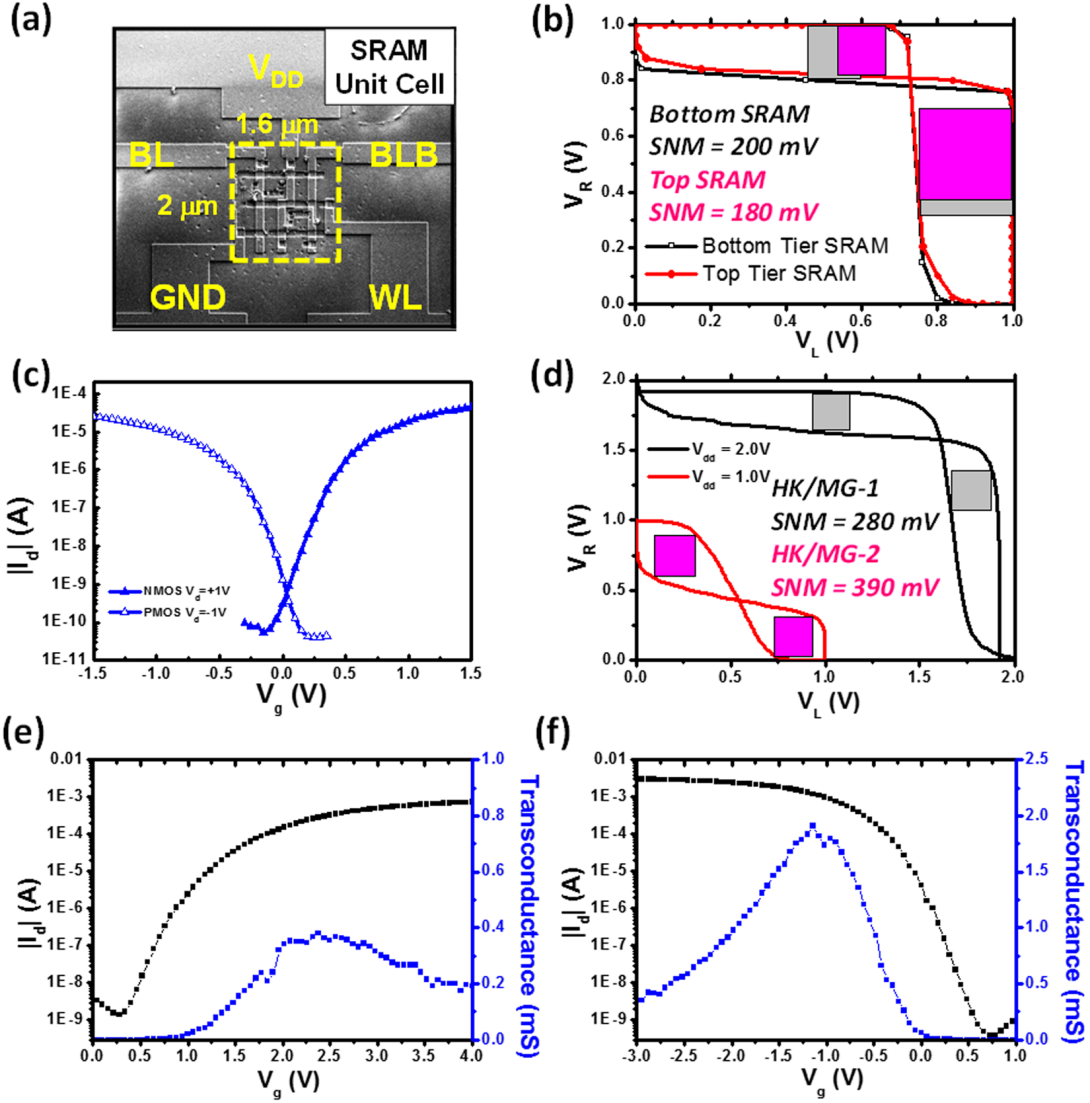
(**a**) A photograph of the compact 6T-SRAM circuit with a footprint of 2.0 × 1.6 µm^2^. (**b**) A butterfly curve of bottom tier SRAM circuit and top tier SRAM circuit, which is used to determine SNM. (**c**) The improved transfer characteristics of threshold-voltage-optimized gate structures by HfO_2_ gate dielectrics and TaN metal gate deposition. (**d**) An improved butterfly curve threshold-voltage-optimized gate structures, yielding a larger SNM. (**e** and **f**) Transfer characteristics and transconductance performance of multi-channel NMOSFETs and PMOSFETs for TFTs, analog or RF circuits implementation.

For demonstration of circuits from the 3D-IC, static random access memory (SRAM) cell was used as a test vehicle for a basic CMOS circuit integration and the CMP 20 nm was selected as the thickness of the final active layer based on experimental results. Figure 5(a) shows the image of stackable six transistors-SRAM (6T-SRAM) cell structure composed of two PMOSFETs and four NMOSFETs with a channel length of 50 nm in a compact footprint of 2.0 × 1.6 µm^2^. Static noise margins (SNMs) are widely used as the criteria of store stability evaluation where the traditional butterfly plot is mostly used^33^. The performance of two SRAM circuits was measured as shown in Fig. 5(b), of which static noise margin (SNM) are 180 and 200 mV for top and bottom tier SRAM cells at a supply voltage (V_DD_) of 1.0 V, respectively. As discussed in previous section, the bottom tier circuits has one more time exposed to the laser irradiation so that the SNM performance (Opened dot curve in Fig. 5b) will be slightly improved, comparing to performance of top tier circuits (Closed dot curve in Fig. 5b). Note that the above structure is the gate level stacking. An alternative of transistor level stacking, namely transistors on transistors, can more effectively utilize chip area with less mask process^34^. Further footprint reduction can be achieved by re-design of layout where two PMOSFETs of SRAM cells are stacked on four NMOSFETs of SRAM cell (Figure S9). This smaller footprint 6 T SRAM bit-cell based on the monolithic 3D IC sequential integration possess exhibits at least 25% footprint saving. However, the SNM performance of Fig. 5(b) is still not good enough because of non-symmetry threshold voltages at +0.18 V and −0.10 V for NMOSFETs and PMOSFETs as shown in Fig. 5(c). Therefore, the tuning of HK/MG structure is also a critical factor and they still exhibit enhanced current as high as 181/188 μA/μm (|V_d_| = 1 V and |V_g_| = 1 V) with steep SS of 107/98 mV/dec for NMOSFETs and PMOSFETs (Fig. 5c), respectively. The SNM performance of the V_th_ optimized SRAM is shown in Fig. 5(d) whose the HfO_2_-gate-dielectric-based SRAM exhibits a maximum SNM of 390 mV at a supply voltage (V_DD_) as low as 1.0 V as compared to the Al_2_O_3_-gate-dielectric-based SRAM with a maximum SNM of 280 mV at a supply voltage (V_DD_) of 2 V, demonstrating a low power consumption circuit.

Moreover, to further meet the requirement of high on-current and transconductance (G_m_) for future demand of TFTs, multi-channel structure TFTs based on analog or RF circuits are also demonstrated in this work. Based on the better gate control ability with a small gate length (~180 nm), multi-channel layouts were designed by dividing single wide channel to splitting narrow channels. The stability of SRAM circuit depends on the static noise margin and a better SNM performance will be obtained by the better symmetric threshold voltages of MOSFETs and larger gain of CMOS inverters. Figure 5(e) and (f) show transfer characteristics of multi-channel NMOSFETs and PMOSFETs, and the corresponding parameters are summarized in Table 3. Obviously, the better performance such as the lower subthreshold swing (S.S.) and the higher field effect mobility (µ_FE_) can be still observed in PMOSFETs. In addition, maximum transconductance and saturation current of multi-channel NMOSFETs and PMOSFETs (I_d,sat_) are 0.39, 1.91 mS and 731, 1350 µA at |V_g_| = 2 V with an identical on/off ratio (I_on_/I_off_) of ~10^6^, respectively. Although the ratio is not very high because the thinner EOT results in higher GIDL current, it is an acceptable value for such specific circuits.

**Table 3:** Transfer characteristics of multi-channel NMOSFETs and PMOSFETs.

W = 10 µm	S.S. (mV/dec)	V_th_ (V)	G_m_ (mS)	I_on_/I_off_	μ_FE_ (cm^2^/V-s)	I_d,sat_ (∝A)
L = 180 nm						
NMOSFET	160	0.82	0.39	5.11 × 10^5^	6.04	731
PMOSFET	142	0.11	1.91	7.74 × 10^6^	30.30	1350

## Conclusions

In summary, a novel methodology to fabricate TFTs based MOSFETs and SRAM circuits with high performance and low power consumptions were reported. By combining solid state laser crystallization process, CMP planarization and CO_2_ laser activation, sub-50 nm TFTs based MOSFETs, showing the high on-current/low S.S. of 181 µA/µm/107 mV/dec for NMOSFETs and 188 µA/µm/98 mV/dec for PMOSFETs as well as stackable SRAM with SNM = 390 mV at V_DD_ = 1.0 V, were demonstrated. Furthermore, high-performance TFTs fabricated by all the low thermal budget process (<400 °C) were also demonstrated through the implementation of the monolithic 3D-IC circuits. The 6T-SRAM cell in low-voltage operation was demonstrated with the better stability while the advances in the process technology are helpful to improve area usage efficiency. This advanced 3D processed architecture with closely spaced ILD enables high-performance stackable MOSFETs and SRAM for power-saving IoT/mobile products on low cost or flexible substrate.

## Methods

### Deposition of an a-Si film followed by a Nd:YAG laser (λ = 532 nm) crystallization process and CMP planarized poly-Si thin film

All the thin films fabrication were established on a 8-inch wafer. First, a box SiO_2_ layer (500 nm) for device isolation was deposited by plasma enhanced chemical vapor deposition (PECVD) using SiH_4_ as precursor. Subsequently, an a-Si active layer was deposited by high-density plasma chemical vapor deposition (HDP-CVD) with a maximum process temperature of 400 °C where the high-density plasma is helpful to reduce the defect density for devices channel implementation and free of conventional high temperature (500 °C) dehydrogenation process^1, 2^. The thickness of a-Si layers are designed as 20 nm, 50 nm and 150 nm (denoted as LC 20 nm, LC 50 nm and LC 150 nm, respectively) for following laser crystallization process. A solid state (Nd:YAG crystal with a tuned wavelength of 532 nm via a frequency doubler) pulsed laser was used to crystallize a-Si thin films to poly-Si thin films with a scan speed of 6 cm/s, fixed repetition rate of 50 kHz, pulsed width of 15 ns and an energy density of 320 mJ/cm^2^, respectively. A 150 nm-thick a-Si film exhibits the largest grain size for the following devices fabrication. Standard CMP planarization methodology with specific etching slurry for Si material was used to reduce the thickness of the poly-Si thin films to 120 nm, 50 nm and 20 nm (denoted as CMP 120 nm, CMP 50 nm and CMP 20 nm, respectively). Thin film characterizations of these samples are described as below.

### Dopant activation process in NMOSFETs and PMOSFETs by CO_2_ far-infrared laser annealing technology and device fabrication of CMOS compatible 6T-SRAM circuits

All the devices fabrication were established on a 8-inch complementary metal-oxide-semiconductor (CMOS) compatible process line. E-beam lithography (EBL) was used to define patterns of devices and CMOS circuit structures with a minimum size of sub-50 nm. Devices sizes are defined as 50 nm, 70 nm, 400 nm, 1 µm and 10 µm as the channel width (also length) by EBL, where the gate dielectric structure, Al_2_O_3_/TiN (HK/MG-1), was achieved by plasma enhanced atomic layer deposition (PEALD) and physical vapor deposition (PVD), respectively. Implantation of source and drain (S/D) regions are As and BF_2_ dopants with doses of 5 × 10^15^ ions/cm^2^ and an energy of 10 keV for NMOSFETs and PMOSFETs, respectively. CO_2_ far infrared pulsed laser activation (CO_2_-FIR-LA) with a wavelength of 10.6 µm with a scan speed of 6 cm/s, fixed repetition rate of 10 kHz, pulse duration of 1 µs and a power of 140 W was used to activate dopants by the implantation induced defect absorption mechanism. Note that the thermal budget of CO_2_-FIR-LA is 400 °C (substrate heater) to enhance defect absorption. After the contact hole etching process, a nickel silicide (NiSi) process with a maximum temperature of 400 °C was used to decrease the contact resistance. Back-end metallization with two metal layers (M2) was designed to reduce the footprint of typical six transistors static random-access memory (6T-SRAM) circuits. Typical SRAM cell layout is fairly dense because the most of the contacts (Bitline, V_DD_ and Gnd) are shared. To meet the requirement of the low supply voltage (low power consumption) circuit, a flat band voltage with an optimized gate structure of HfO_2_/TaN (HK/MG-2) is also integrated to NMOSFETs, PMOSFETs and SRAM circuits.

### Characterizations and Measurements

Surface morphologies including average grain size and average roughness of laser crystallized a-Si thin films and CMP planarized poly-Si thin films were performed by field emission scanning electron microscopy with an accelerated voltage of 15 keV (FESEM, JSE-6500F) and atomic force microscope (AFM, Veeco Dimension 5000 Scanning Probe Microscope). Microstructure orientations were measured from grazing incidence X-ray diffraction (GIXRD, PAN analytical X’pert pro) analysis with a Cu target source (Kα, λ = 0.154 nm). The analysis conditions of 2θ scan are from 20°–80° with a step size of 0.01° at an incident angle (omega) of 1°. GIXRD is a common technique for the study of crystal structures and atomic spacing. Thus, the thin film residual stress could be extracted from XRD-sin^2^ψ technique given by (*d*_*ϕψ*_ − *d*_*0*_)/*d*_*0*_ = [(*1* + *ν*)/*E*]*σ_ϕ_*sin^*2*^ *ψ* *−* (*ν*/*E*)*(*σ*_*1*_ + *σ*_*2*_). Hall measurements with a van der Pauw configuration were used to extract intrinsic mobility and carrier concentration. Transfer characteristics (I_d_-V_g_ curve) and output characteristics (I_d_-V_d_, not shown here) were measured using three probes configuration in the probe station to determine subthreshold swing, threshold voltage and on-current. The temperature of 25 ± 1 °C was actively controlled during measurements. Field effect mobility (μ_FE_) and effective mobility (μ_eff_) in surface-inversion layers, including were thus extracted by *μ*_*FE*_ = (*L*/*W*)(*g*_*m*_/*C*_ox_*V_*d*_) and *μ*_*eff*_ = (*L*/*W*)(*g*_*D*_/*C*_*ox*_(*V*_*g*_ − *V*_*th*_)) = (*L*/*W*)(*g*_*D*_/*Q*_*inv*_). Butterfly curves for SRAM circuits were measured using six probes configuration in the probe station and corresponding static noise margins (SNM) were defined as the length of the side of largest square that can be embedded inside the lobes of the butterfly curve.

## Electronic supplementary material

Supplementary information
