# Supplementary material for: High Performance and Low power Monolithic Three-Dimensional Sub-50 nm Poly Si Thin film transistor (TFTs) Circuits
Source: Sci Rep. 2017 May 2;7:1368. doi: 10.1038/s41598-017-01012-y (PMC5431052; doi:10.1038/s41598-017-01012-y)
Supplement: Supplementary file 1 — Supplementary information [file 41598_2017_1012_MOESM1_ESM.doc]

**Supplementary Information**

**High Performance and Low power Monolithic Three-Dimensional Sub-50 nm Poly Si Thin film transistor (TFTs) Circuits**

*Tsung-Ta Wu^a^, Wen-Hsien Huang,^a^ Chih-Chao Yang,^a^ Hung-Chun Chen,^a^ Tung-Ying Hsieh,^a^ Wei-Sheng Lin,^b^ Ming-Hsuan Kao,^c^ Chiu-Hao Chen,^a^ Jie-Yi Yao,^a^ Yi-Ling Jian,^a^ Chiung-Chih Hsu,^a^ Kun-Lin Lin,^a^ Chang-Hong Shen, ^a^* Yu-Lun Chueh ^b^* and Jia-Min Shieh ^a^**

*^a^* National Nano Device Laboratories, No.26, Prosperity Road 1, Hsinchu, 30078, Taiwan.

*^b^* Department of Materials Science and Engineering, National Tsing Hua University, Hsinchu 30013, Taiwan.

*^c^* Departments of Photonics and Institute of Electro-Optical Engineering, National Chiao-Tung University, Hsinchu 30010, Taiwan.

E-mail: ylchueh@mx.nthu.edu.tw, chshen@narlabs.org.tw and jmshieh@narlabs.org.tw

**Figure S1** Schematic and comparison of TSV parallel integration and monolithic 3D-IC

**Figure S2** AFM image for thin films roughness of laser crystallized poly-Si thin films at different original a-Si thickness and CMP planarized poly-Si thin films.

**Figure S3** XRD analysis spectra of laser crystallized and CMP planarized poly-Si thin films.

**Table S1** XRD analysis summary for crystallinity, orientation and lattice strain for laser crystallized and CMP planarized poly-Si thin films.

| **LC 150 nm** | **FWHM** | **Net area** | **Intensity Ratio %** | **Lattice Strain %** |
| --- | --- | --- | --- | --- |
| (111) | 0.38 | 623 | 62.87 | **0** |
| (220) | 0.39 | 368 | 37.13 | **0** |
| Total | | 991 | 100.00 |  |

| **LC 50 nm** | **FWHM** | **Net area** | **Intensity Ratio%** | **Lattice Strain%** |
| --- | --- | --- | --- | --- |
| (111) | 0.47 | 191 | 64.09 | **0.476** |
| (220) | 0.47 | 107 | 35.91 | **0.260** |
| Total | | 298 | 100.00 |  |

| **LC 20 nm** | **FWHM** | **Net area** | **Intensity Ratio%** | **Lattice Strain%** |
| --- | --- | --- | --- | --- |
| (111) | 0.68 | 32 | 41.03 | **0.973** |
| (220) | 1.57 | 46 | 58.97 | **1.502** |
| Total | | 78 | 100.00 |  |

| **CMP 120 nm** | **FWHM** | **Net area** | **Intensity Ratio%** | **Lattice strain%** |
| --- | --- | --- | --- | --- |
| (111) | 0.38 | 454 | 60.53 | **0** |
| (220) | 0.39 | 296 | 39.47 | **0** |
| Total | | 750 | 100.00 |  |

| **CMP 50 nm** | **FWHM** | **Net area** | **Intensity Ratio%** | **Lattice strain%** |
| --- | --- | --- | --- | --- |
| (111) | 0.41 | 208 | 58.26 | **0.265** |
| (220) | 0.41 | 149 | 41.74 | **0.125** |
| Total | | 357 | 100.00 |  |

| **CMP 20 nm** | **FWHM** | **Net area** | **Intensity Ratio%** | **Lattice strain%** |
| --- | --- | --- | --- | --- |
| (111) | 0.52 | 73 | 52.52 | **0.609** |
| (220) | 0.56 | 66 | 47.48 | **0.399** |
| Total | | 139 | 100.00 |  |

***Calcualtions of residual stress :***

Another important feature of residual stress can be measured by using various techniques including X-ray differaction or wafer curvature.^1^ The XRD-sin^2^*ψ* technique from the theories of crystallography and solid mechanics for thin film residual stress extraction is used by the limited penetration of X-rays in solid surfaces at a series of fixed incidence angles (omega = 1.0^o^) and over a pre-defined diffraction angle (2θ = 20^o^ – 80^o^). The principal formula for the XRD-sin^2^*ψ* stress measurement can be written as,

*(d_φψ_ - d_0_)/d_0_ = [(1+ν)/E]*σ_φ_*sin^2^ψ−(ν/E)*(σ_1_ + σ_2_)*

where the term of *(d_φψ_ - d_0_)/d_0_* represents the lattice strain (it is also shown in Table S1) and *d_0_* is the strain-free spacing (LC 150nm condition), *ν* and *E* are Poisson‘s ratio and Young’s modulus of Si (denoted as 0.2209 and 162.0 Gpa, respectively), *ψ* is defined as the angle between surface normal and diffraction plane normal and *σ_1_ / σ_2_ *are the two principal stress components of in-plane stress *σ_φ_*. Therefore, the obtained 2θ values from Bragg’s diffraction were used to plot the linear regression in order to obtain the slope (*[(1+ν)/E]*σ_φ_*) and used for the stress (*σ_φ_*) calculation.^2-4^ (linear regression results with CMP planarization in Figure S4)

**Figure S4** Linear regression results of CMP planarized poly-Si thin films for stress calculation.

**Figure S5** Conventional TFTs performance with the channel width/length of 10 μm/10 μm.

**Table S2.** Parameters of channel width / length (100 µm / 100 µm) for NMOSFETs and PMOSFETs as the function of channel thicknesses.

| **NMOSFET**  **W/L=10 μm/10 μm** | **S.S. (mV/dec)** | **V_th_ (V)** | **I_on_ (uA/um)** | **N_it_ (cm^-2^)** | **μ_FE_ (cm^2^/V-s)** |
| --- | --- | --- | --- | --- | --- |
| **LC 150 nm** | **235** | **1.83** | **3.6** | **22.3 x 10^12^** | **17.8 ±3.4** |
| **CMP 120 nm** | **279** | **2.43** | **1.5** | **27.8 x 10^12^** | **20.7 ±5.3** |
| **CMP 50 nm** | **259** | **2.07** | **3.3** | **25.3 x 10^12^** | **22.5 ±3.5** |
| **CMP 20 nm** | **253** | **2.08** | **1.8** | **24.5 x 10^12^** | **12.5 ±2.4** |

| **PMOSFET**  **W/L=10 μm/10 μm** | **S.S. (mV/dec)** | **V_th_ (V)** | **I_on_ (uA/um)** | **N_it_ (cm^-2^)** | **μ_FE_ (cm^2^/V-s)** |
| --- | --- | --- | --- | --- | --- |
| **LC 150 nm** | **134** | **0.28** | **2.5** | **9.5 x 10^12^** | **30.7 ±3.2** |
| **CMP 120 nm** | **259** | **-0.02** | **3.5** | **25.3 x 10^12^** | **23.7 ±7.5** |
| **CMP 50 nm** | **161** | **0.34** | **8.7** | **12.8 x 10^12^** | **19.6 ±2.7** |
| **CMP 20 nm** | **150** | **0.10** | **9.7** | **11.5 x 10^12^** | **21.1 ±2.8** |

**Figure S6** Devices effective mobility as a function of gate voltage.

**Figure S7** Schematic of CO_2_ far-infrared laser activation (CO_2_-FIR-LA) mechanism.

**Figure S8** Exhibition of uniformity laser activated devices performance.

**Figure S9** Representative optimized SRAM layout based on transistor-level stacking.

**References**

1. Luo, Q. & Jones, A.H. High-precision determination of residual stress of polycrystalline coatings using optimised XRD-sin^2^ψ technique. *Surf. Coat. Technol.*, **205**, 1403-1408 (2010).
2. Janssen, G.C.A.M. Stress and strain in polycrystalline thin films. *Thin Solid Films*, **515**, 6654-6664 (2007).
3. Scardi, P., Polonioli, P. & Ferrari, S. Residual stress in stabilized zirconia thin films prepared by r.f. magnetron sputtering. *Thin Solid Films*, **253**, 349-355 (1994).
4. Teixeira, V., Andritschky, M., Fischer, W., Buchkremer, H.P. & Stover, D. Effects of deposition temperature and thermal cycling on residual stress state in zirconia-based thermal barrier coatings. *Surf. Coat. Technol.*, **120-121**, 103-111 (1999).
